# HIF-1α expression in liver metastasis but not primary colorectal cancer is associated with prognosis of patients with colorectal liver metastasis

**DOI:** 10.1186/s12957-020-02012-5

**Published:** 2020-09-07

**Authors:** Yuma Wada, Yuji Morine, Satoru Imura, Tetsuya Ikemoto, Yu Saito, Chie Takasu, Shinichiro Yamada, Mitsuo Shimada

**Affiliations:** Department of Surgery, Institute of Health Biosciences, The University of Tokushima Graduate School, 3-18-15, Kuramoto-cho, Tokushima, 770-8503 Japan

**Keywords:** Colorectal liver metastasis, HIF-1α, Prognostic factor, Molecular biology

## Abstract

**Background:**

The role of hypoxia-inducible factor-1α (HIF-1α) in primary colorectal cancer (CRC) and colorectal liver metastasis (CRLM) has remained unclear. The aim of this study was to investigate HIF-1α expression and its association with prognosis in patients with CRLM with a focus on hepatic stellate cells (HSCs).

**Methods:**

Colon cancer cells were cultured in HSC-conditioned medium (CM), and HIF-1α expression and cell migration were analyzed. Seventy-five patients with CRLM who underwent an initial curative hepatectomy were enrolled. We examined HIF-1α expressions and patient prognosis between primary CRCs and the matched liver metastatic specimens.

**Results:**

Activated HSCs induced HIF-1α mRNA and protein expression in colon cancer cells (*p* < 0.01) and promoted cell migration (*p* < 0.01). The positive rates of HIF-1α expression in primary CRCs and liver metastases were 68.0 and 72.0%, respectively. There were no differences in overall (OS) and disease-free survival (DFS) of HIF-1α expression in primary CRC. However, HIF-1α expression in liver metastasis correlated to poor prognosis in both OS and DFS. Furthermore, patients with HIF-1α positive expression in liver metastasis had poor prognosis.

**Conclusion:**

HIF-1α expression in liver metastasis determines poor prognosis of CRLM patients. HSCs might play a key role in aggressive phenotypes of tumor cells.

## Background

In patients with colorectal liver metastasis (CRLM), surgical resection improves patient prognosis and is recommended if the resection will be curative [1–5]. On the other hand, with the implementation of “targeted” molecular therapies against epidermal growth factor receptor (EGFR) and vascular endothelial growth factor (VEGF), the median overall survival (OS) of patients with metastatic colorectal cancer (CRC) has progressively improved, surpassing 30 months [6–8]. The monoclonal antibodies like cetuximab, panitumumab, and bevacizumab have demonstrated effectiveness, both in terms of better response and improved survival. A comparison of various markers between primary and metastatic tumors have been recently reported [9–13]. Previous studies reported that while the immune microenvironment in the primary CRC tumor and liver metastasis is different, hypoxia-inducible factor-1α (HIF-1α) expression in primary CRC was comparable to that in corresponding metastases and HIF-1α expression is consistent in primary CRC and matched metastatic tissues [9, 10]. However, whether primary CRC and the associated metastases have similar molecular features remains unknown. A comparative analysis between primary CRC and metastatic tumors may improve understanding of the various molecular alterations in metastatic tumors and facilitate research and development of novel targeted drugs for CRC.

Once cancer cells in the primary tumor site migrate to distant metastatic sites, some cells around the tumor cells, such as sinusoidal endothelial cells, macrophages, or fibroblasts, can increase tumor malignancy [14]. In the liver microenvironment, hepatocytes, Kupffer cells, and hepatic stellate cells (HSCs) play an important role [15–17]. In the liver cancer microenvironment, Kupffer cells and HSCs are activated by cancer cells, and tumor-associated macrophages (TAMs) and activated HSCs regulate tumor malignant behavior [17]. HSCs play a key role in the development of aggressive phenotypes of tumor cells. We previously reported that activated HSCs promoted cancer cell progression through paracrine or autocrine interleukin-6 (IL-6) [18]. However, there are few reports about the relationship between activated HSCs, which are considered cancer-associated fibroblasts, and metastatic cancer cells in CRLM. We speculate that the characteristics of metastatic cancer cells could be modified by cancer-associated fibroblasts in the cancer microenvironment of liver.

The HIF-1α transcription factor [19–23] plays a central role in biologic processes under hypoxic conditions including angiogenesis [24, 25], tumor growth [26], and epithelial mesenchymal transition [27] in several cancer types. A previous report showed that high HIF-1α expression correlated to tumor malignancy in liver compared with some metastatic organs such as bone and lung [28]. Moreover, another report showed that HIF-1α expression was altered from primary sites to metastatic sites, and high expression of HIF-1α in the metastatic site correlated to poor prognosis [29]. Therefore, HIF-1α expression might serve a critical role to regulate tumor malignancy in CRLM.

The aim of this study was to elucidate a possible mechanism of activated HSCs on augmenting tumor malignancy and to investigate the association of HIF-1α expression between primary CRC and liver metastasis on CRLM patient prognosis.

## Methods

### In vitro study

#### Cell culture

The HCT116 colon cancer cell line was obtained from the Riken Cell Bank, and the hepatic stellate cell line LX2 was obtained from Cellular Engineering Technologies Inc. HCT116 cells were cultured in McCoy’s 5A Modified Medium (Life Technologies Ltd., Tokyo, Japan) with 10% fetal bovine serum (FBS) (Life Technologies Ltd.). LX2 cells were cultured in Dulbecco’s modified Eagle’s medium (DMEM) (Life Technologies Ltd.) with 10% FBS. Both cell lines were cultured under 37 °C in 5% CO_2._

#### HSC conditioned (CM) medium preparation

HCT116 cells (3.0 × 10^6^ cells) were cultured in McCoy medium in a 10-cm dish until cell numbers reached 3.0 × 10^5^ cells. The cell culture media was then changed to CM from cancer cells for 24 h of culture. The medium of HCT116 (3.0 × 10^6^ cells) changed to activated HSCs conditioned medium (HSC-CM) or DMEM (control) for 24 h culture. After that, the FBS-free medium culture was followed. After 24 h, HCT116 cells were collected for experimental analyses.

#### Scratch assays

Cells were plated in 6-cm dishes at 3.0 × 10^6^ cells/dish. The medium was replaced with activated HSC-CM or DMEM (control) for 24 h. After the cells had reached confluency, a plastic pipette tip was drawn across the center of the plate to produce a scratch that was 1 mm in width. After 24 h of culture in medium with 1% FBS, a phase contrast microscope was used to examine cell movement into the wound area.

#### Polymerase chain reaction (PCR) analysis

RNA was extracted from samples using the RNeasy Mini Kit (Qiagen, Hilden, Germany) according to the manufacturer’s instructions. cDNA was synthesized using a reverse transcription kit (Applied Biosystems, Foster City, CA, USA). The HIF-1α TaqMan gene expression assay (Hs00153153_m1, Applied Biosystems) was used, and GAPDH (4326317E, Applied Biosystems) was selected as an internal control. The StepOnePlus Real-Time PCR System (Applied Biosystems) was used to perform qRT-PCR.

#### Western blotting

RIPA buffer (Thermo Fisher Scientific Inc.) containing both protease inhibitor cocktail (Sigma-Aldrich, St Louis, MO, USA) and the PhosSTOP phosphatase inhibitor cocktail (Roche, Tokyo, Japan) was used to lyse cells. Protein concentrations were measured with the BCA kit (Thermo Fisher Scientific Inc.), and equal amounts of extracted proteins were separated on 10% SDS-PAGE gels and transferred onto PVDF membranes (Bio-Rad Inc., Hercules, CA, USA). The membranes were incubated with the indicated primary antibody, followed by the appropriate HRP-conjugated secondary antibody. The bands were detected by chemiluminescence (Thermo Fisher Scientific Inc.). Primary antibody against HIF-1α (diluted 1:1000; HPA001275) was purchased from Sigma-Aldrich, and primary antibody against β-actin was obtained from Sigma Chemical (St Louis, MO, USA).

### Clinicopathological study

#### Patient selection

Seventy-five CRLM patients who underwent an initial hepatectomy at our institute from 1994 to 2015 with available surgical specimens of primary CRCs and the matched liver metastases were enrolled in this study. This study was authorized in advance by the Institutional Review Board of the University of Tokushima Graduate School (approval ID number: 2392), and all patients provided written informed consent. The participants in this study included 47 males and 28 females with a mean age of 66.5 years, ranging from 33 to 90 years in age. The numbers of patients with synchronous and metachronous liver metastases were 32 (43%) and 43 (57%), respectively. Staging and curability was defined according to the Classification of Primary Colorectal Cancer by the Colorectal Cancer Study Group of Japan [30]. T-factor was determined by tumor number, size, and vascular infiltration. Tumor stage was determined by T-, N-, and M-factors. We defined H class as the following classification: H0 class, No metastasis to liver; H1 class, ≦ 4 lesions and ≦ 5 cm; H2 class, other than H1 and H3; H3 class, > 5 lesions and > 5cm [31]. We divided surgical procedure into minor and major hepatectomy, and major hepatectomy was defined as resection of four or more liver segments [32]. All patients had not received neoadjuvant chemotherapy and follow-up period had started after hepatectomy. The mean follow-up period was 41.3 months (range 4.4–191.3 months). We examined clinicopathological features, prognosis, molecular biological malignancy, 5-year overall survival (OS), and disease-free survival (DFS).

#### Immunohistochemical assessment of HIF-1α

**Fig. 1:**
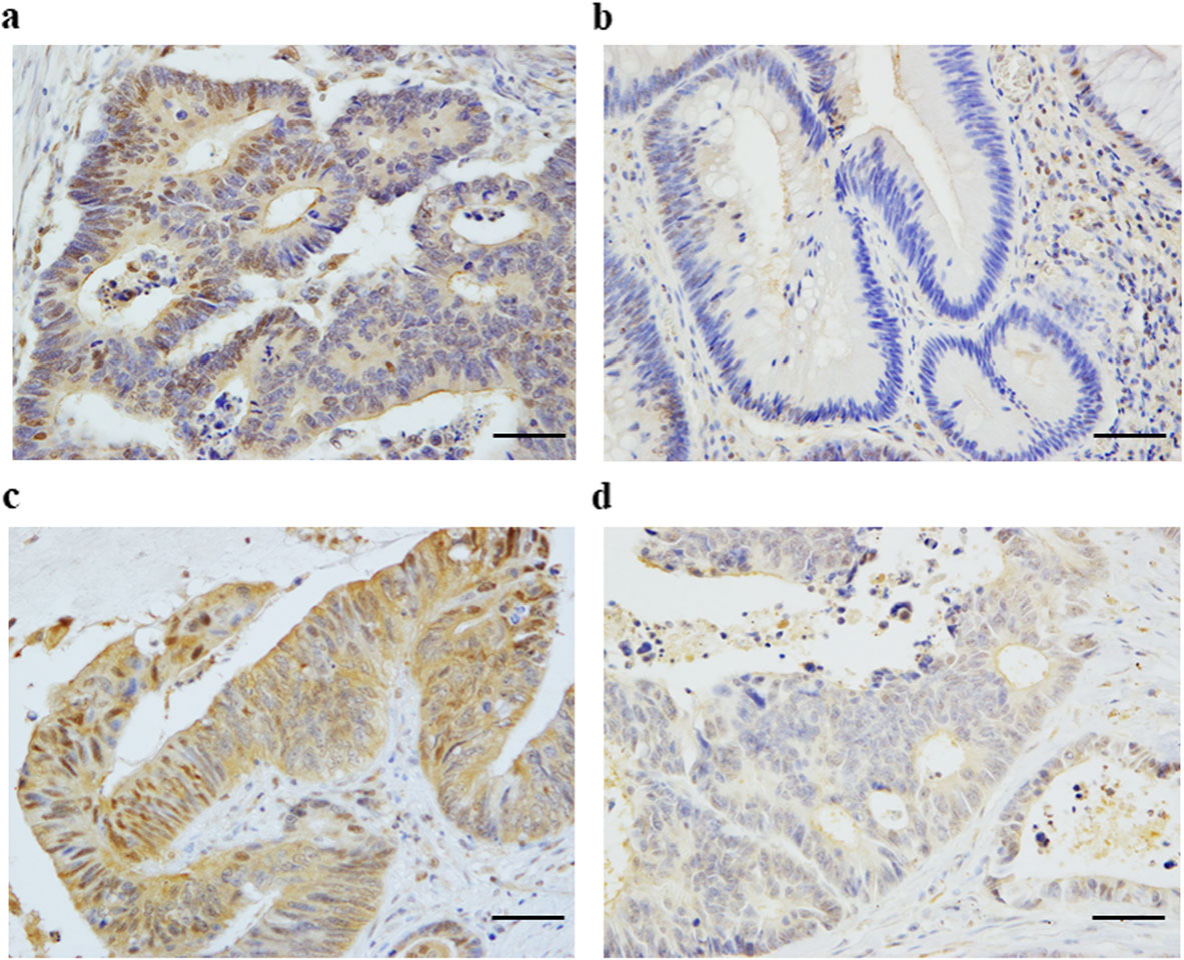
Immunohistochemical staining of HIF-1α. **a** High expression of HIF-1α in a primary tumor, **b** low expression of HIF-1α in a primary tumor, **c** high expression of HIF-1α in a liver metastasis, and **d** low expression of HIF-1α in a liver metastasis. Scale bar, 100 μm. Magnification, × 400

Paraffin sections (4 μm) were cut from archival formalin-fixed paraffin-embedded tissue blocks. The samples were deparaffinized and dehydrated using a graded series of ethanol solutions. Endogenous peroxidase activity was stopped through the administration of 0.3% hydrogen peroxidase and methanol for 20 min. After rinsing in phosphate-buffered saline (PBS; Fisher Scientific, Pittsburgh, PA, USA), the tissue sections were processed in a 0.01 M citrate buffer (pH 6.0) inside a heat-resistance plastic container. The sections were irradiated in a microwave oven for 25 min and then allowed to cool at room temperature. The sections were incubated with primary mouse monoclonal antibody against HIF-1α (1:500; HPA001275, Sigma-Aldrich, MO, USA) overnight at 4 °C in a humidified chamber. The sections were incubated using Daco REAL^TM^ Envision^TM^/HRP, Rabbit/Mouse (ENV), for 45 min followed by three washes in PBS. Peroxidase labeling was developed by incubating the section in 3,3′-diaminobenzidine tetrahydrochloride (DAB) for 5 min. Nuclear counterstaining was completed using Mayer’s hematoxylin solution. Cell counts were performed using a Nikon Digital Camera DXM 1200F photomicroscope at a magnification of × 200 (× 20 objective and × 10 eyepiece). The area counted in each section was randomly selected from the representative tumor field. For each section, eight areas were assessed. The staining score for HIF-1α was determined based on staining intensity (0 negative, 1 low, 2 medium, 3 high) and staining area (0, 0%; 1, 0–25%; 2, 26–50%; 3, ≥ 51%). Scores over 4 points were defined as positive expression (Fig. 1).

### Statistics

All statistical analyses were performed using statistical software (JMP software, version 11; SAS Campus Drive, Cary, NC, USA). Data are expressed as the mean ± SD. Survival curves were calculated using the Kaplan–Meier method and compared using the log-rank test. Comparisons between two groups were performed by Mann–Whitney *U* test. Comparisons between more than three groups were calculated using one-way ANOVA with Turkey–Kramer’s test. *p* < 0.05 was considered to indicate statistical significance.

## Results

### Tumor malignancy is enhanced in the liver by HSCs

**Fig. 2:**
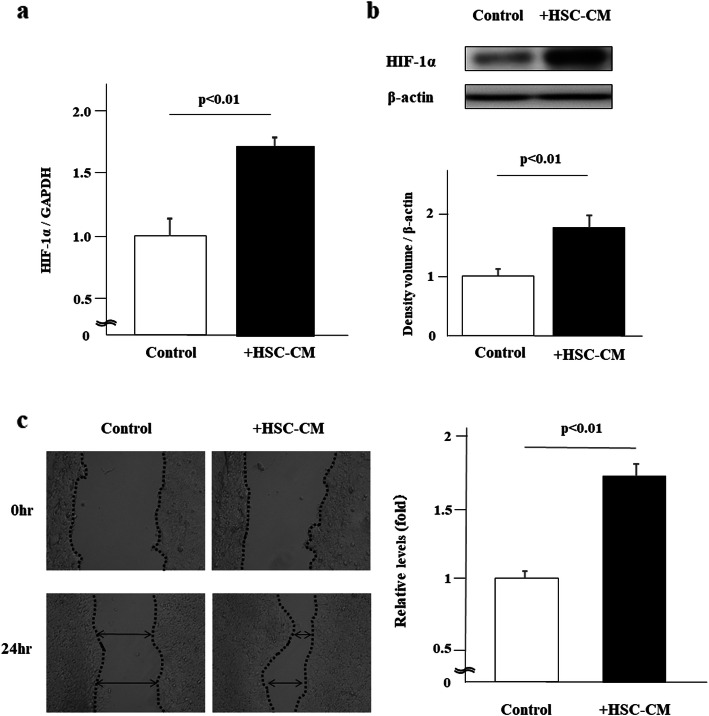
Hepatic stellate cells (HSCs) increased HIF-1α expression in HCT116 colorectal cancer cells. HSCs promoted cancer cell migration. HCT116 colorectal cancer cells were co-cultured with HSC conditioned medium (HSC-CM). **a** PCR analysis for HIF-1α mRNA expression in cancer cells and **b** western blot analysis for HIF-1α expression in cancer cells (*n* = 4). **c** Scratch assays were performed and cells were examined after 24 h (*n* = 4). *P < 0.01 compared with controls

We first examined the impact of HSC-CM on HIF-1α expression in HCT116 colorectal cancer cells. We found that HSC-CM induced both HIF-1α mRNA and protein expression in HCT116 cells (*p* < 0.01) (Fig. 2a, b). Moreover, HSC-CM induced migration of HCT116 cancer cells in scratch assays (*p* < 0.01) (Fig. 2c). Together, these results suggest the possibility that cells in the cancer microenvironment such as HSCs may alter tumor malignancy in liver metastasis.

### HIF-1α expression in liver metastasis determines patient prognosis

We next examined HIF-1α expression in 75 CRLM patients with primary CRC and matched liver metastasis specimens. Positive HIF-1α expression was detected in 51 (68.0%) primary CRCs and 54 (72.0%) liver metastases. We observed the following trends of HIF-1α expression from primary CRC to liver metastasis: positive to positive expression, 37 (49.3%); positive to negative expression, 14 (18.7%); negative to positive expression, 17 (22.7%); and negative to negative expression, 7 (9.3%). Tables 1 and 2 show the patient clinicopathological factors according to HIF-1α expression in primary and metastatic sites. There were no significant relationships between HIF-1α high and low expression groups in both primary and metastatic sites. The adjuvant chemotherapy was introduced in 45 cases (60%) of all patients. According to detail regimen of the chemotherapy, we performed 5-FU-based chemotherapy in most of patients and showed the detail as follows: FOLFOX, 8 cases; LV/UFT, 8 cases; LV/5FU, 7 cases; MMC/5FU, 7 cases; IRIS, 7 cases; arterial injection. 3 cases; oral 5FU, 2 cases; and other, 3 cases.

**Table 1:** Clinicopathological factors according to HIF-1α expression in metastatic site

Factors		Low expression (*n* = 21)	High expression (*n* = 54)	*p* value
Primary factors				
Location	Colon/rectum	12/9	34/20	0.6433
Depth	< SS/≥ SS	2/19	5/49	0.9718
Diff.	Diff/undiff	20/1	51/3	0.8896
Lymphatic invasion	−/+	9/12	20/33	0.6614
Vessel invasion	−/+	5/16	17/37	0.5068
LN metastasis	−/+	8/13	24/30	0.6165
Metastatic factors				
Meta. period	Meta/syn	10/11	22/32	0.5895
Tumor size (cm)	< 5/≥ 5	15/6	42/12	0.5678
Tumor number	< 5/≥ 5	17/4	43/11	0.8973
H class	H1/H2.3	12/9	34/20	0.6433
Grade	A/B.C	12/9	26/28	0.4836
Adjuvant therapy	−/+	8/13	22/32	0.8337
Hepatectomy	Minor/major	16/5	46/8	0.3555
CA19-9^a^	< 100/≥ 100	16/4	43/4	0.2014
CEA^a^	< 10/≥ 10	12/8	27/20	0.8461

*Diff* differentiated type, *undiff* undifferentiated type, *LN* lymph node, *SS* subserous, *Meta* metachronous, *Syn* synchronous

^a^8 patients are not available

**Table 2:** Clinicopathological factors according to HIF-1α expression in primary site

Factors		Low expression (*n* = 24)	High expression (*n* = 51)	*p* value
Primary factors				
Location	Colon/rectum	16/8	30/21	0.5130
Depth	< SS/≥ SS	1/23	6/45	0.2601
Diff.	Diff/undiff	23/1	48/3	0.7528
Lymphatic invasion	−/+	10/14	19/31	0.6476
Vessel invasion	−/+	6/18	16/35	0.5684
LN metastasis	−/+	9/15	23/28	0.5334
Metastatic factors				
Meta. period	Meta/syn	7/17	25/26	0.1005
Tumor size (cm)	< 5≥ 5	18/6	39/12	0.8896
Tumor number	< 5/≥ 5	19/5	41/10	0.9018
H class	H1/H2.3	14/10	32/19	0.7150
Grade	A/B.C	11/13	27/24	0.5656
Adjuvant therapy	−/+	8/16	22/29	0.4188
Hepatectomy	Minor/major	17/7	45/6	0.1633
CA19-9^a^	< 100/≥ 100	19/1	40/7	0.2196
CEA^a^	< 10/≥ 10	10/10	29/18	0.3758

*Diff* differentiated type, *undiff* undifferentiated type, *LN* lymph node, *SS* subserous, *Meta* metachronous, *Syn* synchronous

^a^8 patients are not available

**Fig. 3:**
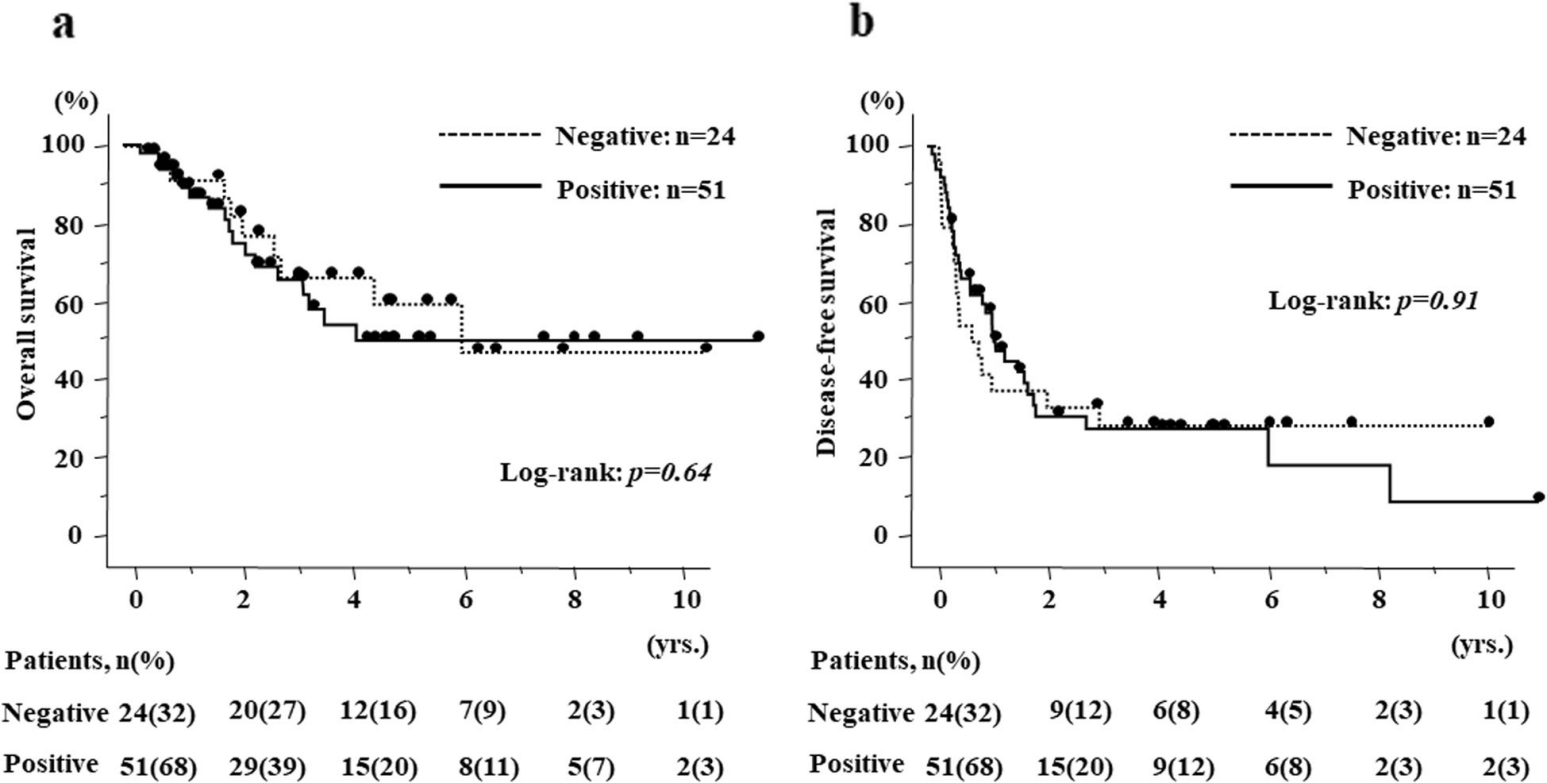
Kaplan–Meier curves according to HIF-1α expression in primary site of CRLM. **a** Overall survival curves according to HIF-1α expression and **b** disease-free survival curves according to HIF-1α expression

**Fig. 4:**
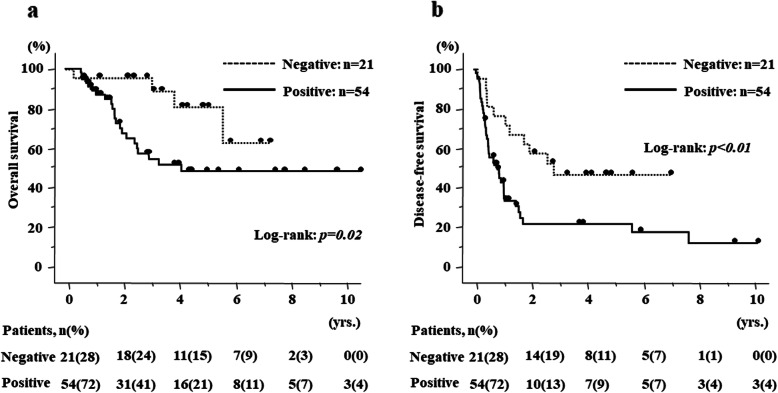
Kaplan–Meier curves according to HIF-1α expression in metastatic site of CRLM. **a** Overall survival curves according to HIF-1α expression and **b** disease-free survival curves according to HIF-1α expression

**Fig. 5:**
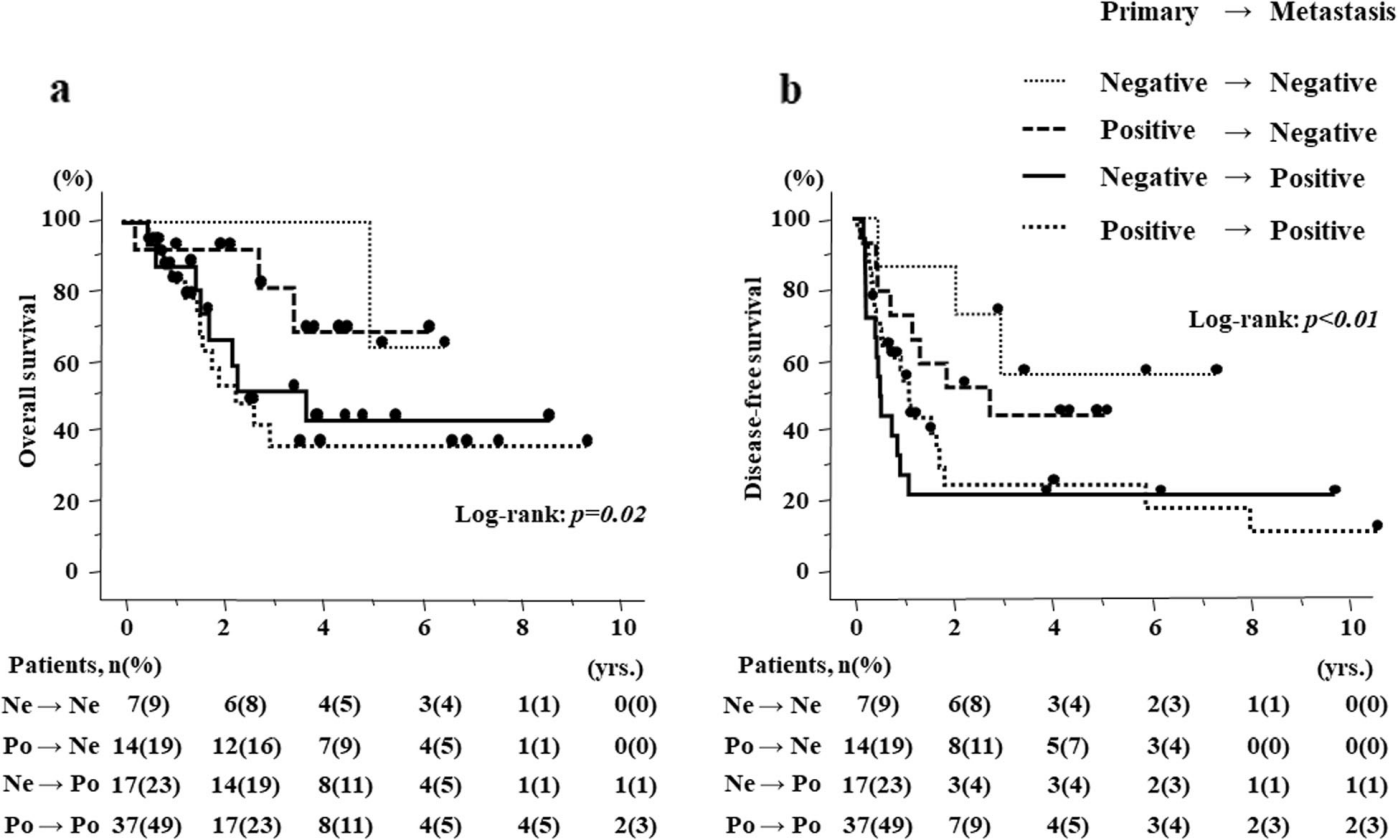
Kaplan–Meier curves according to the alteration of HIF-1α expression from primary CRC to liver metastasis in CRLM. **a** Overall survival curves according to the alteration of HIF-1α expression and **b** disease-free survival curves according to the alteration of HIF-1α expression

Regarding survival, there were no differences in OS and DFS according to HIF-1α expression in the primary site (*p* = 0.64 (95%CI 0.55–2.85), *p* = 0.91 (95%CI 0.56–1.83), respectively) (Fig. 3a, b). The percentage of patients according to HIF-1α expression (Low/High) in the primary site were 67.2/69.7% and 60.4/54.6% for 3 and 5 years in OS, and 45.8/62.2%, 33.3/31.2% for 1 and 3 years in DFS, respectively. In contrast, HIF-1α expression in the metastatic site significantly correlated with poor prognosis in both OS and DFS (*p* = 0.02 (95%CI 1.24–11.17), *p* < 0.01 (95%CI 1.30–5.16), respectively) (Fig. 4a, b). The percentage of patients according to HIF-1α expression (Low/High) in the metastatic site were 95.2/58.6% and 75.8/54.9% for 3 and 5 years in OS and 71.4/51.1% and 52.2/19.6% for 1 and 3 years in DFS. More importantly, in 41.3% of patients, HIF-1α expression was altered from primary CRC to liver metastasis, and the patients with positive HIF-1α expression in liver metastasis had significantly poor prognosis (Fig. 5a, b).

Univariate analysis of OS revealed that differentiation type (undifferentiated type, *p* < 0.0001) in primary tumors and H class (2.3, *p* = 0.0400), grade (B.C, *p* = 0.0119), and HIF-1α expression (positive, *p* = 0.0220) in metastatic tumors were significant prognostic factors (Table 3). In multivariate analysis, undifferentiated type (HR 20.873, *p* = 0.0013) in primary tumors and high HIF-1α expression (HR 2.850, *p* = 0.0422) in metastatic tumors were independent prognostic factors (Table 3).

**Table 3:** Prognostic factors of overall survival

Factors	5-year survival (%)		Univariate		Multivariate	
			HR (95% CI)	*p* value	HR (95% CI)	*p* value
Primary factors						
Location	Colon/rectum	56.0/58.0	1.03 (0.46–2.24)	0.9357		
Depth	< SS/> SS	65.6/58.9	0.93 (0.28–5.81)	0.9242		
Diff.	Diff/undiff	59.1/0	23.90 (4.58–112.20)	*< 0.0001*	20.87 (3.78–107.13)	*0.0013*
Lymphatic invasion	−/+	66.5/53.7	0.93 (0.43–2.13)	0.9569		
Vessel invasion	−/+	59.9/56.7	1.16 (0.53–2.75)	0.7172		
LN metastasis	−/+	70.3/48.7	1.53 (0.70–3.59)	0.3019		
HIF-1α	−/+	60.4/54.6	1.21 (0.55–2.85)	0.6424		
Metastatic factors						
Meta. period	Meta/syn	69.0/49.8	1.79 (0.79–4.59)	0.1826		
Tumor size (cm)	< 5/> 5	60.5/47.2	1.85 (0.81–4.04)	0.1213		
Tumor number	< 5/> 5	58.4/51.1	1.67 (0.68–3.72)	0.2231		
H class	H1/H2.3	63.8/46.0	2.22 (1.02–4.96)	*0.0400*	1.19 (0.41–4.31)	0.7591
Grade	A/B.C	72.7/44.2	2.89 (1.27–7.42)	*0.0119*	2.37 (0.20-0.61)	0.1996
Adjuvant therapy	−/+	68.3/54.4	1.22 (0.53–3.13)	0.6514		
Hepatectomy	Minor/major	52.6/64.7	0.68 (0.20–1.79)	0.4832		
HIF-1α	−/+	75.8/54.9	3.26 (1.24–11.17)	*0.0220*	2.85 (1.04–10.01)	*0.0422*
CA19-9	< 100/> 100	28.2/37.5	0.73 (0.27–2.53)	0.2557		
CEA	< 10/> 10	57.4/55.1	1.45 (0.60–3.84)	0.4164		

*Diff* differentiated type, *undiff* undifferentiated type, *LN* lymph node, *SS* subserous, *Meta* metachronous, *Syn* synchronous, *HR* hazard ratio, *CI* confidence interval

Univariate analysis of DFS revealed that differentiation type (undifferentiated type, *p* = 0.0466) and lymph node metastases (positive, *p* = 0.0146) in primary tumors and grade (B.C, *p* = 0.0119) and HIF-1α expression (positive, *p* = 0.0073) in metastatic tumors were significant recurrent factors (Table 4). In multivariate analysis, lymph node metastases (HR 2.03, *p* = 0.0186) in primary tumors and grade B.C (HR 2.21, *p* = 0.0057) and high HIF-1α expression (HR 2.40, *p* = 0.0079) in metastatic tumors were independent recurrent factors (Table 4).

**Table 4:** Prognostic factors of disease-free survival

Factors	3-year survival (%)		Univariate		Multivariate	
			HR (95% CI)	*p* value	HR (95% CI)	*p* value
Primary factors						
Location	Colon/rectum	27.3/30.7	1.17 (0.67–2.10)	0.4980		
Depth	< SS/> SS	18.8/31.2	0.77 (0.23–1.91)	0.6370		
Diff.	Diff/undiff	31.1/0	3.16 (1.00–8.92)	*0.0466*	2.14 (0.50–6.27)	0.2650
Lymphatic invasion	−/+	32.6/31.0	1.05 (0.58–1.87)	0.7784		
Vessel invasion	−/+	28.0/32.7	0.92 (0.50–1.64)	0.9157		
LN metastasis	−/+	50.7/18.4	1.91 (1.07–3.51)	*0.0146*	2.03 (1.12–3.79)	*0.0186*
HIF-1α	−/+	33.3/31.2	1.03 (0.56–1.83)	0.9122		
Metastatic factors						
Meta. period	Meta/syn	39.6/23.8	1.71 (0.97–3.13)	0.0693		
Tumor size (cm)	< 5/> 5	26.1/41.7	0.95 (0.52–1.86)	0.8708		
Tumor number	< 5/> 5	30.8/27.8	1.58 (0.79–2.94)	0.1647		
H class	H1/H2.3	31.3/27.9	1.55 (0.88–2.70)	0.1224		
Grade	A/B.C	37.6/22.7	2.02 (1.16–3.58)	*0.0119*	2.21 (1.26–3.94)	*0.0057*
Adjuvant therapy	−/+	30.2/23.8	1.54 (0.96–3.34)	0.1681		
Hepatectomy	Minor/major	28.8/46.2	0.97 (0.44–1.92)	0.9361		
HIF-1α	−/+	52.2/19.6	2.48 (1.30–5.16)	*0.0073*	2.40 (1.25–5.01)	*0.0079*
CA19-9	< 100/> 100	63.5/37.5	1.71 (0.74–4.98)	0.5631		
CEA	< 10/> 10	27.6/30.1	1.19 (0.66–2.20)	0.5739		

*Diff* differentiated type, *undiff* undifferentiated type, *LN* lymph node, *SS* subserous, *Meta* metachronous, *Syn* synchronous, *HR* hazard ratio, *CI* confidence interval

## Discussion

The present study revealed that activated HSCs increased HIF-1α expression at the mRNA and protein levels and promoted tumor cell activities. The positive rate of HIF-1α expression was similar in primary CRC (68.0%) and liver metastasis (72.0%). Nevertheless, in 41.3% of patients, HIF-1α expression was altered from primary CRC to liver metastasis, and the patients with positive HIF-1α expression in liver metastasis had significantly poor prognosis. HIF-1α expression in primary CRC did not influence any malignant behavior including prognostic outcome.

Previous studies reported differences in the immune microenvironment between the primary CRC and liver metastasis, and more CD33+ cells and CD8+ cells, but not CD8+ T cells in liver metastases; these results suggested that increased numbers of immunosuppressing cells in the liver may contribute to the poor response to immunotherapy [9]. The presence of liver metastases was associated with fewer infiltrating CD8+ T cells and poor response to PD-1 therapy in other cancer types [33]. CRC-associated DNA hypomethylation undergoes hypermethylation in liver metastases [11]. Therefore, immune cells and epigenetic modifications might correlate to the alteration of positive HIF-1 expression in liver metastasis and poor prognosis of patients with positive HIF-1 expression in liver metastasis.

In the present study, undifferentiated type in primary tumors and HIF-1α high expression in metastatic tumors were the independent prognostic factors in OS. As the independent recurrent factor, lymph node metastases in primary factors and synchronous, grade B.C, and high HIF-1α expression in metastatic tumors were observed. Previous studies reported that lymph node metastases, lymphovascular invasion, and poorly differentiated type in primary tumors and the number of liver metastases were independent poor prognostic factors for progression-free survival and OS in CRLM patients [34–36]. The incidence of synchronous metastasis remains high with poor survival outcomes compared with patients of metachronous metastasis [37].

Hepatocytes, Kupffer cells, and HSCs are important cells in the microenvironment of liver. In CRLM, Kupffer cells and HSCs are activated by cancer cells, and these activated stromal cells interact with metastatic cancer cells and promote tumor invasion [17]. As we previously reported that activated HSCs promote cancer cell progression through paracrine or autocrine IL-6 [18], it is known that activated HSC regulate downstream pathways and promote tumor growth [38, 39]. Our further research revealed a correlation between IL-6 expression and HIF-1α expression, but there is no significantly difference (data not shown). As our supportive opinion, other reports showed that exosomes from activated HSCs induce HIF-1α expression and affect the metabolic switch of liver nonparenchymal cells [40]. Another report showed that tamoxifen decreases the levels of HIF-1α expression by suppressing activated HSC [41]. However, as it is still unknown how activated HSC induced HIF-1α expression in cancer cell, further studies on the relationship of HSC and HIF-1α are required.

To our knowledge, this is the first study focusing on HSCs in the microenvironment and comparing HIF-1α expression between primary CRC and liver metastases. However, this study had some limitations, including its retrospective design and the small sample size. Furthermore, as we do not have the data of period from surgery to the beginning of adjuvant chemotherapy, we were not able to compare chemotherapy response with the alterations of HIF-1α expression. Our results only demonstrated a relationship between HSCs and cancer cells. Since other important cells are present in the cancer microenvironment such as TAMs and immune cells, further research on these cells is required. Our results suggest that HIF-1α expression in liver metastasis is not associated with the primary CRC and may be a useful prognostic marker. These findings should be confirmed in future studies.

## Conclusions

HIF-1α expression in liver metastasis, but not primary CRC, is correlated with poor prognosis of patients with CRLM, and HSCs might play a key role in the aggressive phenotype of tumor cells. These findings may improve our understanding of various molecular alterations in metastatic tumors and guide the development of novel targeted drugs.

## Data Availability

Not applicable.

## Abbreviations

CRC: Colorectal cancer
CRLM: Colorectal liver metastasis
HSC: Hepatic stellate cell
CAF: Cancer-associated fibroblast
HIF-1α: Hypoxia-inducible factor-1α
OS: Overall survival
DFS: Disease-free survival
TAM: Tumor-associated macrophage
EMT: Epithelial mesenchymal transition
CM: Conditioned medium
IL-6: Interleukin-6

## References

[1] Choti MA, Sitzmann JV, Tiburi MF, Sumetchotimetha W, Rangsin R, Schulick RD (2002). Trends in long-term survival following liver resection for hepatic colorectal metastases. Ann Surg..

[2] Cummings LC, Payes JD, Cooper GS (2007). Survival after hepatic resection in metastatic colorectal cancer: a population-based study. Cancer..

[3] Kavlakoglu B, Ustun I, Oksuz O, Pekcici R, Ergocen S, Oral S (2011). Surgical treatment of liver metastases from colorectal cancer: experience of a single institution. Arch Iran Med..

[4] Michael GH, Hiromichi I, Mithat G, Yuman F, Peter JA, Ronald PD (2010). Survival after hepatic resection for metastatic colorectal cancer: trends in outcomes for 1600 patients during two decades at a single institution. J Am Coll Surg..

[5] Aloia TA, Vauthey JN, Loyer EM, Ribero D, Pawlik TM, Wei SH (2006). Solitary colorectal liver metastasis: resection determines outcome. Arch Surg..

[6] Jensen NF, Smith DH, Nygård SB, Rømer MU, Nielsen KV, Brünner N (2012). Predictive biomarkers with potential of converting conventional chemotherapy to targeted therapy in patients with metastatic colorectal cancer. Scand J Gastroenterol..

[7] Welch HG, Robertson DJ (2016). Colorectal cancer on the decline—why screening can’t explain it all. N Engl J Med..

[8] Cremolini C, Schirripa M, Antoniotti C, Moretto R, Salvatore L, Masi G (2015). First-line chemotherapy for mCRC—a review and evidence-based algorithm. Nat Rev Clin Oncol..

[9] Zhou SN, Pan WT, Pan MX, Luo QY, Zhang L, Lin JZ, et al. Comparison of immune microenvironment between colon and liver metastatic tissue in colon cancer patients with liver metastasis. Dig Dis Sci. 2020. 10.1007/s10620-020-06203-8. 10.1007/s10620-020-06203-8 32193860

[10] Yin L, Li J, Ma D, Li D, Sun Y (2020). Angiogenesis in primary colorectal cancer and matched metastatic tissues: biological and clinical implications for anti-angiogenic therapies. Oncol Lett..

[11] Orjuela S, Menigatti M, Schraml P, Kambakamba P, Robinson MD, Marra G (2020). The DNA hypermethylation phenotype of colorectal cancer liver metastases resembles that of the primary colorectal cancers. BMC Cancer..

[12] Wu JB, Sarmiento AL, Fiset PO, Lazaris A, Metrakos P, Petrillo S (2019). Histologic features and genomic alterations of primary colorectal adenocarcinoma predict growth patterns of liver metastasis. World J Gastroenterol..

[13] Kim EK, Song MJ, Jung Y, Lee WS, Jang HH (2019). Proteomic analysis of primary colon cancer and synchronous solitary liver metastasis. Cancer Genomics Proteomics..

[14] Quail DF, Joyce JA (2013). Microenvironmental regulation of tumor progression and metastasis. Nat Med..

[15] Eggert T, Greten TF (2017). Tumor regulation of the tissue environment in the liver. Pharmacol Ther..

[16] Affo S, Yu LX, Schwabe RF (2017). The role of cancer-associated fibroblasts and fibrosis in liver cancer. Annu Rev Pathol..

[17] Pnina B (2016). Role of the microenvironment in liver metastasis: from pre-to prometastatic niche. Clin Cancer Res..

[18] Iwahasi S, Rui F, Morine Y, Yamada S, Saito YU, Ikemoto T (2020). Hepatic stellate cells contribute to the tumor malignancy of hepatocellular carcinoma through the IL-6 pathway. Anticancer Res..

[19] Zhong H, De Marzo AM, Laughner E, Lim M, Hilton DA, Zagzag D (1999). Overexpression of hypoxia-inducible factor 1alpha in common human cancers and their metastases. Cancer Res..

[20] Birner P, Schindl M, Obermair A, Breitenecker G, Oberhuber G (2001). Expression of hypoxia-inducible factor 1alpha in epithelial ovarian tumors: its impact on prognosis and on response to chemotherapy. Clin Cancer Res..

[21] Sumiyoshi Y, Kakeji Y, Egashira A, Mizokami K, Orita H, Maehara Y (2006). Overexpression of hypoxia-inducible factor 1alpha and p53 is a marker for an unfavorable prognosis in gastric cancer. Clin Cancer Res..

[22] Batmunkh E, Shimada M, Morine Y, Imura S, Kanemura H, Arakawa Y (2010). Expression of hypoxia-inducible factor-1 alpha (HIF-1alpha) in patients with the gallbladder carcinoma. Int J Clin Oncol..

[23] Miyake K, Yoshizumi T, Imura S, Sugimoto K, Batmunkh E, Kanemura H (2008). Expression of hypoxia-inducible factor-1alpha, histone deacetylase 1, and metastasis-associated protein 1 in pancreatic carcinoma: correlation with poor prognosis with possible regulation. Pancreas..

[24] Griffiths EA, Pritchard SA, Valentine HR, Whitchelo N, Bishop PW, Ebert MP (2007). Hypoxia-inducible factor-1a expression in the gastric carcinogenesis sequence and its prognostic role in gastric and gastro-esophageal adenocarcinomas. Br J Cancer..

[25] Kim Y, Nam HJ, Lee J, Park DY, Kim C, Yu YS (2016). Methylation-dependent regulation of HIF-1a stability restricts retinal and tumor angiogenesis. Nat Commun..

[26] Pouysségur J, Dayan F, Mazure NM (2006). Hypoxia signaling in cancer and approaches to enforce tumor regression. Nature..

[27] Huihui L, Matjaz R, Longchang J, David H, Heiko H (2017). Antagonistic effects of p53 and HIF1A on microRNA-34a regulation of PPP1R11 and STAT3 and hypoxia-induced epithelial to mesenchymal transition in colorectal cancer cells. Gastroenterology..

[28] Dupuy F, Tabariès S, Andrzejewski S, Dong Z, Blagih J, Annis MG (2015). PDK1-dependent metabolic reprogramming dictates metastatic potential in breast cancer. Cell Metabolism..

[29] Shimomura M, Hinoi T, Kuroda S, Adachi T, Kawaguchi Y, Sasada T (2013). Overexpression of hypoxia inducible factor-1 alpha is an independent risk factor for recurrence after curative resection of colorectal liver metastasis. Ann Sug Oncol..

[30] Hashiguchi Y, Muro K, Saito Y, Ito Y, Ajioka Y, Hamaguchi T (2020). Japanese Society for Cancer of the Colon and Rectum (JSCCR) guidelines 2019 for the treatment of colorectal cancer. Int J Clin Oncol..

[31] Yamaguchi T, Mori T, Takahashi K, Matsumoto H, Miyamoto H, Kato T (2008). A new classification system for liver metastases from colorectal cancer in Japanese multicenter analysis. Hepatogastroenterology..

[32] Reddy SK, Barbas AS, Turley RS, Steel JL, Tsung A, Marsh JW (2011). A standard definition of major hepatectomy: resection of four or more liver segments. HPB (Oxford)..

[33] Tumeh PC, Hellmann MD, Hamid O, Tsai KK, Loo KL, Gubens MA (2017). Liver metastasis and treatment outcome with anti-pd-1 monoclonal antibody in patients with melanoma and NSCLC. Cancer Immunol Res..

[34] Li Destri G, Barchitta M, Pesce A, Latteri S, Bosco D, Di Cataldo A (2019). Predictive value of the number of harvested lymph nodes and cut-off for lymph node ratio in the prognosis of stage II and III colorectal cancer patients. J Invest Surg..

[35] Yonemura K, Kajiwara Y, Ao T, Mochizuki S, Shinto E, Okamoto K (2019). Prognostic value of poorly differentiated clusters in liver metastatic lesions of colorectal carcinoma. Am J Surg Pathol..

[36] Jang KU, Kim CW, Kim KH, Lim SB, Yu CS, Kim W (2016). Prognostic factors in terms of the number of metastatic nodules in patients with colorectal cancer liver metastases. Ann Coloproctol..

[37] Okholm C, Mollerup TK, Schultz NA, Strandby RB, Achiam MP (2018). Synchronous and metachronous liver metastases in patients with colorectal cancer. Dan Med J..

[38] Friedman SL (2008). Mechanisms of hepatic fibrogenesis. Gastroenterology..

[39] Yin C, Evason KJ, Asahina K, Stainier DY (2013). Hepatic stellate cells in liver development, regeneration, and cancer. J Clin Invest..

[40] Wan L, Xia T, Du Y, Liu J, Xie Y, Zhang Y (2019). Exosomes from activated hepatic stellate cells contain GLUT1 and PKM2: a role for exosomes in metabolic switch of liver nonparenchymal cells. FASEB J..

[41] Cortes E, Lachowski D, Rice A, Thorpe SD, Robinson B, Yeldag G (2019). Tamoxifen mechanically deactivates hepatic stellate cells via the G protein-coupled estrogen receptor. Oncogene..

